# Novel European free-living, non-diazotrophic *Bradyrhizobium* isolates from contrasting soils that lack nodulation and nitrogen fixation genes – a genome comparison

**DOI:** 10.1038/srep25858

**Published:** 2016-05-10

**Authors:** Frances Patricia Jones, Ian M. Clark, Robert King, Liz J. Shaw, Martin J. Woodward, Penny R. Hirsch

**Affiliations:** 1 Department of AgroEcology, Rothamsted Research, Harpenden, AL5 2JQ, UK; 2 Department of Geography and Environmental Science, University of Reading, Reading, RG6 6AH, UK; 3 Department of Computational and Systems Biology, Rothamsted Research, Harpenden, AL5 2JQ, UK; 4 Department of Food and Nutritional Sciences, University of Reading, Reading, RG6 6AH, UK

## Abstract

The slow-growing genus *Bradyrhizobium* is biologically important in soils, with different representatives found to perform a range of biochemical functions including photosynthesis, induction of root nodules and symbiotic nitrogen fixation and denitrification. Consequently, the role of the genus in soil ecology and biogeochemical transformations is of agricultural and environmental significance. Some isolates of *Bradyrhizobium* have been shown to be non-symbiotic and do not possess the ability to form nodules. Here we present the genome and gene annotations of two such free-living *Bradyrhizobium* isolates, named G22 and BF49, from soils with differing long-term management regimes (grassland and bare fallow respectively) in addition to carbon metabolism analysis. These *Bradyrhizobium* isolates are the first to be isolated and sequenced from European soil and are the first free-living *Bradyrhizobium* isolates, lacking both nodulation and nitrogen fixation genes, to have their genomes sequenced and assembled from cultured samples. The G22 and BF49 genomes are distinctly different with respect to size and number of genes; the grassland isolate also contains a plasmid. There are also a number of functional differences between these isolates and other published genomes, suggesting that this ubiquitous genus is extremely heterogeneous and has roles within the community not including symbiotic nitrogen fixation.

The slow-growing bacterial genus *Bradyrhizobium* has been shown to be one of the most abundant groups in soil^1^,^2^ including soils sampled from long-term field experiments in the UK (Rothamsted Research, Harpenden)^3^,^4^. A key characteristic of the order Rhizobiales including the genus *Bradyrhizobium* is the ability to form nitrogen-fixing symbioses with legumes to increase nitrogen availability to plants^5^,^6^,^7^,^8^. This ability is thought to have evolved through horizontal gene transfer as the genes involved in this process are usually located on symbiosis islands on the chromosomes of bradyrhizobia^7^,^8^,^9^ or on symbiotic plasmids in many rhizobia^5^. Some isolates of *Bradyrhizobium* have been shown to be non-symbiotic and do not possess the ability to form nodules. The absence of nodulation ability has been noted in the strain *Bradyrhizobium* sp. S23321 isolated from paddy soil in Japan^8^ although nitrogen fixation (*nif*) genes were present. Recently, *Bradyrhizobium* ecotypes from forest soils have been shown to lack both nodulation and nitrogen fixation genes^2^.

*Bradyrhizobium* is biologically important in soils, with different representatives found to perform a wide range of biochemical functions including photosynthesis, nitrogen fixation during symbioses, denitrification and aromatic compound degradation^8^. Nitrogen removal through heterotrophic denitrification is an important step in the global nitrogen cycle carried out by many groups including *Bradyrhizobium*^10^. The multiple roles of *Bradyrhizobium* in the nitrogen cycle make the ecology of this group important for agriculture.

*Bradyrhizobium* is studied extensively due to its symbiotic relationship with soybean and consequently several genomes have been published. Currently, there are seven complete *Bradyrhizobium* genomes in the NCBI database. Six of these are symbiotic and are able to fix nitrogen and form root nodules on legumes (*B. diazoefficiens* USDA 110, *B. japonicum* USDA 6, *B. japonicum* E109, *Bradyrhizobium* sp. ORS278, *Bradyrhizobium* sp. BTAi1 and *B. oligotrophicum* S58) with ORS278, BTAi1 and S58 able to form both stem and root nodules on the aquatic legume *Aeschynomene*^6^,^11^. The other bradyrhizobial genome (*Bradyrhizobium* sp. S23321) is free-living because it is unable to form nodules; however, it still contains the genes required for nitrogen fixation. Four genomes sequenced from North American forest soils were also missing nodulation and nitrogen fixation genes (*Bradyrhizobium* sp. LTSP849, *Bradyrhizobium* sp. LTSP857, *Bradyrhizobium* sp. LTSP885 and *Bradyrhizobium* sp. LTSPM299). These genomes were sequenced using shotgun sequencing of the soil community and were assembled to near completion^2^. Due to the availability of a diverse array of genome reference sequences, *Bradyrhizobium* is an appropriate model to study other soil bacteria: understanding the mechanisms of *Bradyrhizobium* adaptation to independent living in agricultural soils under contrasting management may reveal the genetic potential of this globally important genus.

Here we present the genome and gene annotations and carbon metabolism profiles of two free-living *Bradyrhizobium* isolates from the Highfield experiment at Rothamsted Research that has three long-term treatment regimes: grassland, arable (wheat) and bare fallow tilled regularly to maintain a plant-free soil. Maintenance of these treatments for 60 years has led to distinct differences in soil properties and the soil microbiome^12^. *Bradyrhizobium* sp. G22 and *Bradyrhizobium* sp. BF49 were isolated from soil taken from the permanent grassland and permanent bare fallow plots of the Highfield experiment respectively. These *Bradyrhizobium* strains are the first to be isolated and genome sequenced from European soil and the first free-living and non-diazotrophic isolates, without the presence of either nodulation or nitrogen fixation genes, to have their genomes sequenced and assembled from cultured samples. The isolates were interrogated for differences to determine the level of genetic heterogeneity in carbon metabolism between these isolates.

## Results and Discussion

### General genome description and comparisons

The genome of the grassland isolate G22 is 9,022,917 bp in size while the bare fallow isolate BF49 genome is 7,547,693 bp, constituting a 1.5 Mbp size difference in addition to a 364,482 bp plasmid in G22. The genome size for G22 is similar to nodulating strains *B. diazoefficiens* USDA 110, *B. japonicum* USDA 6, *B. japonicum* E109, *B. oligotrophicum* S58 and *Bradyrhizobium* sp. BTAi1, whereas for BF49 it is closer in size to the free-living strain *Bradyrhizobium* sp. S23321 and the photosynthetic, nodulating strain *Bradyrhizobium* sp. ORS278. Table 1 summarises the genome information of the two novel strains G22 and BF49 along with the other seven completed *Bradyrhizobium* genomes in the database. G22 had 19 contigs which could not be placed in the chromosome or plasmid. These were annotated and used in the subsequent analysis.

G22 shows 1356 more genes (8787) than BF49 (7431) and this rises to 1902 when including the genes contained on the plasmid (541). GC content is similar between the strains at 63.7% and 63.8% for G22 and BF49 respectively, consistent with other *Bradyrhizobium* strains listed in Table 1. The G22 plasmid has a GC content of 60.7%, identical to the plasmid of BTAi1. The G22 and BF49 genomes show high pairwise sequence identity in comparison to both USDA 110 (G22: 84.4%, BF49: 83.4%) and S23321 (G22: 83.4%, BF49: 83.2%) using LASTZ (Large-Scale Genome Alignment Tool).

### Orthologous gene clusters and core genome phylogeny

**Figure 1:**
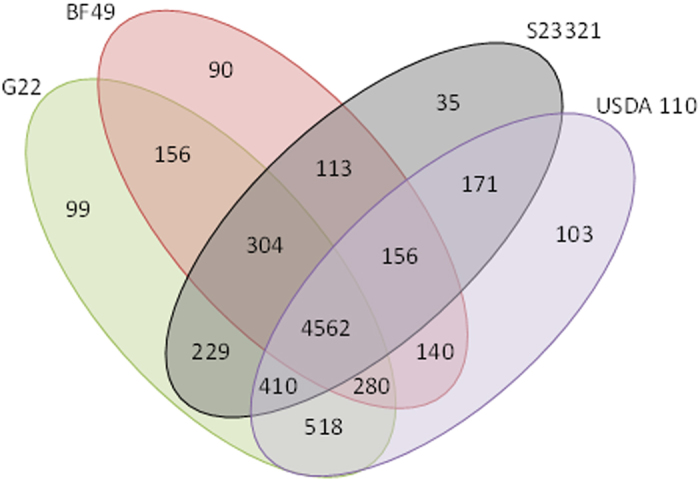
Venn diagram showing orthologous gene clusters for G22, BF49 and two published genomes, S23321 and USDA 110. Output generated using data from OrthoVenn.

**Figure 2:**
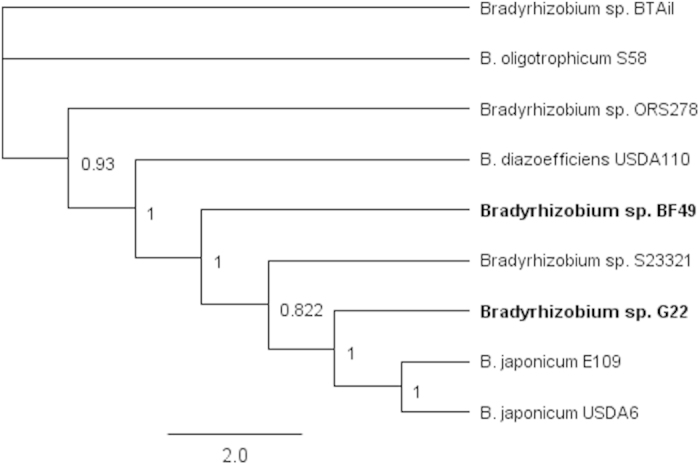
Phylogenetic tree for the 3442 homologous gene clusters in the core genome for the nine *Bradyrhizobium* genomes. The node labels represent the certainty of that node in the phylogenetic tree where one is maximum certainty. Reference sequence: *B. diazoefficiens* USDA 110.

G22 and BF49 were compared with the free-living isolate, S23321, and the symbiotic isolate, USDA 110 (Fig. 1). This suggests that there is a core genome of 4562 genes which are present in all four genomes assessed. The 103 genes present only in the USDA 110 genome include those involved in nodulation and uptake hydrogenase. The 171 genes which are only present in the USDA 110 and S23321 genomes include nitrogen fixation genes. Only a small number of genes are unique to any one isolate (G22: 99, BF49: 90, S23321: 35, USDA 110: 103). OrthoVenn identified a core genome of 3442 homologous gene families across the nine complete genomes available in the database. The core genome SNP analysis (Fig. 2) shows that G22 clusters closest with the *B. japonicum* strains; E109 and USDA 6. BF49 is separate from G22 being more basal. The closest relatives are the free-living strain S23321 and the soybean nodulating type-strain USDA 110. Two of the photosynthetic, *Aeschynomene*-nodulating isolates (BTAi1 and S58) cluster together.

### Isolate identification and 16S phylogeny

**Figure 3:**
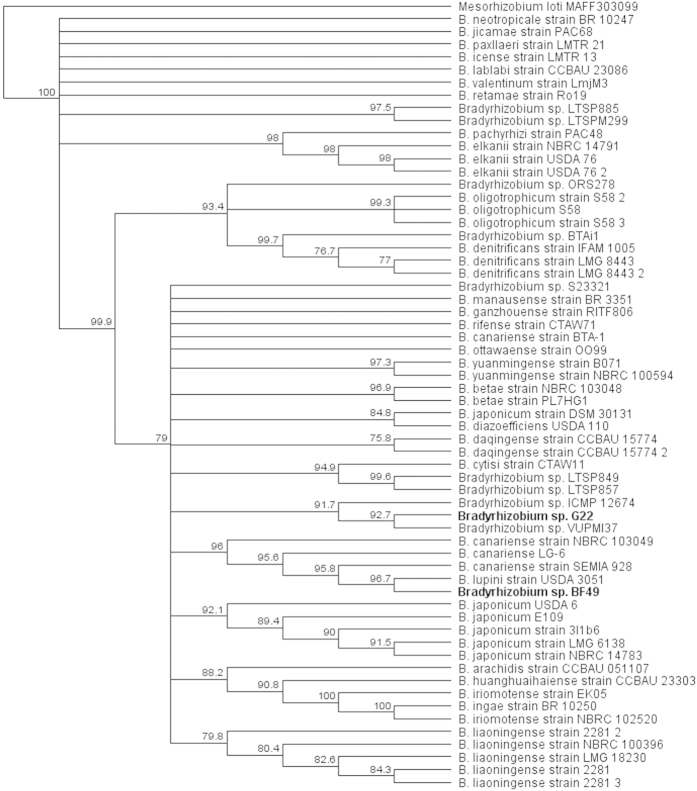
Phylogenetic tree for the 16S rRNA gene using Neighbour-Joining clustering method with 1000 bootstraps.

The 16S rRNA sequence from G22 shows 100% identity with *Bradyrhizobium* sp. VUPMI37 [Accession number: HG940535] and *Bradyrhizobium* sp. ICMP12674 [Accession number: AY491080]^13^ from the NCBI and RDP databases respectively. *Bradyrhizobium* sp. VUPMI37 was originally isolated from *Vigna unguiculata* nodules and *Bradyrhizobium* sp. ICMP12674 was originally isolated from *Ulex europaeus*^13^,^14^. The BF49 16S rRNA fragment shows 100% identity with *B. canariense* SEMIA928 from the NCBI database [Accession number: FJ390904]^15^ and *B. canariense* LG-6 from the RDP database [Accession number: GU306140]. *B. canariense* SEMIA928 was originally isolated from *Lupinus* spp. and *B. canariense* LG-6 was originally isolated from *Lupinus angustifolius* root nodules^16^. The 16S phylogeny (Fig. 3) clusters G22 with VUPMI37 and ICMP12674, which were the top hits from the NCBI and RDP databases. Similarly, BF49 clusters with the top hits from the NCBI and RDP databases (SEMIA928 and LG-6) in addition to *B. lupini* USDA 3051 which was the second hit from the NCBI database [Accession number: NR_134836]. This strain was originally isolated from *Lupinus* and was reclassified from *Rhizobium lupini* to *B. lupini* in 2015^17^. Both G22 and BF49 are in the same clade as the free-living strain, S23321, and the soybean-nodulating strains, E109, USDA 6 and USDA 110. Two of the four strains from North American forest soils, *Bradyrhizobium* sp. LTSP849 and LTSP857^2^ were also in this clade. The photosynthetic strains (ORS278, BTAi1 and S58) are in a separate clade including the remaining two strains from North American forest soils Bradyrhizobium sp. LTSP885 and LTSPM299.

### Genes involved in nitrogen fixation and nodulation

**Figure 4:**
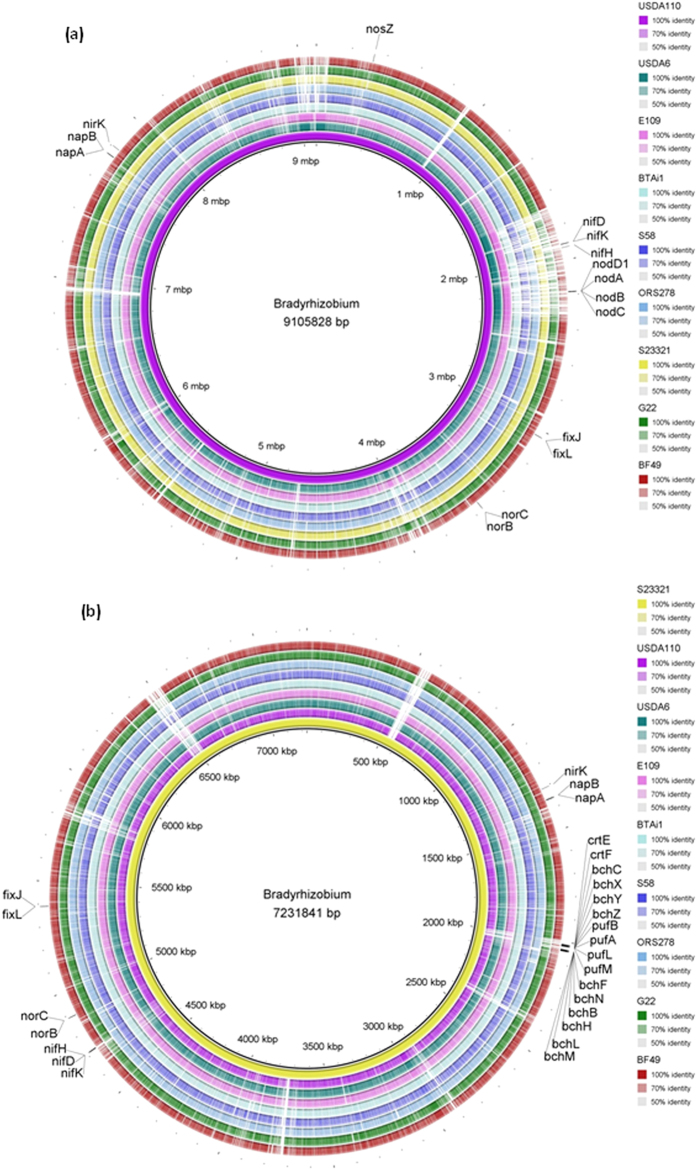
Whole chromosome comparisons between the seven complete *Bradyrhizobium* genomes, BF49 and G22 showing positions of genes involved with nitrogen cycling, nodulation and photosynthesis on the reference genome sequence. Reference sequence: USDA110 (**a**) and S23321 (**b**).

Nitrogen fixation and nodulation genes including *nifDKH, nodD* and *nodABC;* are all absent from both G22 and BF49 (Table 2) and so we suggest that these isolates are not able to either form nodules or fix atmospheric nitrogen. The absence of both *nif* and *nod* genes is in contrast to other published, complete *Bradyrhizobium* genomes and indicates similarity with forest soil bacteria (LTSP849, LTSP857, LTSP885 and LTSPM299)^2^. *B. diazoefficiens* USDA 110 (previously *B. japonicum* USDA 110) is a known symbiotic strain and contains all *nif* and *nod* genes listed. The seven complete *Bradyrhizobium* genomes which have previously been published either contain both *nif* and *nod* genes (the soybean-nodulating strains *B. diazoefficiens* USDA 110, *B. japonicum* USDA 6 and *B. japonicum* E109) or just *nif* genes (the *Aeschynomene-*nodulating strains *Bradyrhizobium* sp. BTAi1, *Bradyrhizobium* sp. S58 and *Bradyrhizobium* sp. ORS278 and the free-living strain *Bradyrhizobium* sp. S23321) (Fig. 4). These strains use a *nod-*independent route for stem and root nodulation of *Aeschynomene*^11^. The FixLJ two component system is present in both the grassland and bare fallow isolate (Table 2). FixLJ acts in response to low oxygen conditions in soil and in the nodule and controls the expression of genes for both nitrogen fixation and denitrification^18^,^19^. This two-component system has also been shown to regulate the response to nitric oxide in *Sinorhizobium meliloti* being shown to regulate a high proportion of genes induced by the presence of nitric oxide^20^. The presence of *fixLJ* in G22 and BF49 is consistent with other *Bradyrhizobium* isolates including all seven completed genomes.

### Genes involved in denitrification

Both genomes encode a nitrate reductase, NapA/B, which catalyses the reduction of nitrate to nitrite; the first stage in denitrification^21^,^22^,^23^. This is common among *Bradyrhizobium* including all seven complete genomes (Table 2 and Fig. 4) and the four genomes from North American forest soils (Table 2). In addition, all fully-sequenced isolates including both G22 and BF49 contain *nirK*, encoding a respiratory copper-containing nitrite reductase which is involved in the second stage of denitrification reducing nitrite to nitric oxide. The third stage of denitrification is the conversion of nitric oxide into nitrous oxide, a potent greenhouse gas, and is catalysed by a nitric oxide reductase encoded by *norB/C*^22^,^23^. The presence of a nitric oxide reductase gene has been noted in all previously published and complete *Bradyrhizobium* genomes in addition to G22 and BF49. The denitrification pathway is not present in the four genomes of strains from North American forest soils.

The ability to convert the greenhouse gas, nitrous oxide into environmentally benign nitrogen gas in the final stage of denitrification is an attribute with global importance^24^. Nitrous oxide reductase encoded by *nosZ* catalyses this process but it is not ubiquitous across *Bradyrhizobium*^22^,^23^,^25^,^26^. Of the seven published complete genomes, only *B. diazoefficiens* USDA 110, *Bradyrhizobium* sp. BTAi1 and *B. oligotrophicum* S58 contain the *nosZ* gene. It is absent from the grassland isolate, G22 but present in the bare fallow isolate, BF49. The presence of the gene shows a potential for BF49 to perform this function and increases the agricultural and environmental importance of this isolate.

### Uptake hydrogenase

The uptake of hydrogen is catalysed by uptake hydrogenase which is encoded by the *hup* genes^27^,^28^,^29^. This process produces ATP which is used by nitrogen-fixing bacteria to mediate for energy lost through the nitrogen fixation process^27^,^29^. The nickel-iron hydrogenase, encoded by *hupSL*^29^, is absent from both G22 and BF49 but is present in all of the symbiotic strains of *Bradyrhizobium* with complete genomes. These genes are also absent from LTSP849, LTSP857, LTSP885 and LTSPM299 (Table 2).

### Photosynthesis and carbon fixation

Genes for photosynthesis are present in four of the published complete *Bradyrhizobium* genomes; S23321 (free-living), S58, BTAi1 and ORS278 (*Aeschynomene* host) (Fig. 4b). These include genes for bacteriochlorophyll (*bchCXYZ*/*FNBHLM*), carotenoids (*crtEF*), light harvesting polypeptides (*pucBAC*/*pufBA*) and reaction centre subunits (*puhA*/*pufLM*)^30^,^31^. They are not present in the soybean-nodulating isolates (USDA 110, USDA 6, and E109), G22 or BF49 genomes or the four isolates from forest soils (LTSP849, LTSP857, LTSP885 and LTSPM299) (Table 2). Many heterotrophic bacteria including *Bradyrhizobium* can fix carbon using the Calvin-Benson-Bassham cycle (CBB cycle). The significance of this is unclear in most cases although the photosynthetic *Aeschynomene*-nodulating strain ORS278 has been shown to require an active CBB cycle for symbiotic nitrogen fixation^32^. The first stage of the CBB cycle is catalysed by Ribulose-1,5-bisphosphate carboxylase oxygenase (RuBisCo) which is present in both G22 and BF49 genomes, consistent with other *Bradyrhizobium* isolates (Table 2). Transketolase is an important enzyme in both the CBB cycle and pentose phosphate pathway, catalysing the interconversion of sugars^33^,^34^. Genes for the transketolase enzyme are present in both G22 and BF49 consistent with all other complete *Bradyrhizobium* isolates which have been published.

### Carbon metabolism

**Figure 5:**
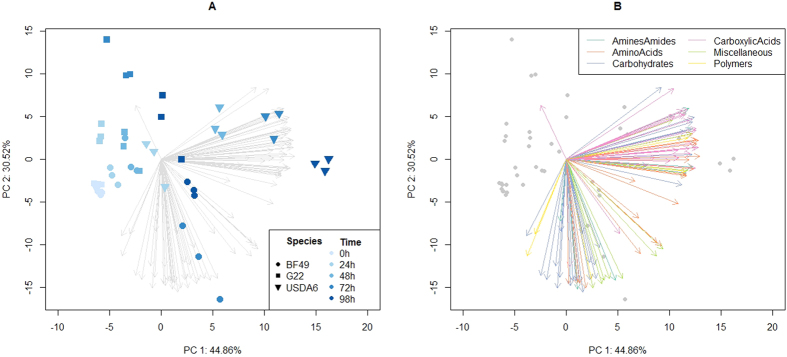
Principal components analysis biplot showing PC1 and PC2 accounting for 75.38% of the variation. (**A**) shows individual replicates across the 5 time points and (**B**) shows the 95 substrates classified into 6 categories.

The principal components analysis (PCA) biplot (Fig. 5A) shows that there is a separation of the time points and USDA 6 from G22 and BF49 across PC1 which accounts for 44.86% of the variation. Across PC2, G22 and BF49 separate and PC2 accounts for 30.52% of the total variation. The first two principal components were visualised as together they accounted for 75.38% of the variation. The third PC accounted for 7.5%. Figure 5B shows the 95 substrates colour coded according to category. More carboxylic acids and amino acid substrates are associated with the separation across PC1; USDA 6 from G22 and BF49. Carbohydrates tend to have a negative direction across PC2 being more closely associated with BF49. One carboxylic acid, malonic acid, was the only substrate which has a positive direction for PC2 and negative for PC1 associating more closely with G22.

Malonic acid can be found in plant tissues, including legumes being first characterised from alfalfa leaves in 1925 and has been found to be in very high concentrations in soybeans^35^,^36^,^37^. This pathway was found to be closely associated with the symbiotic nitrogen fixation pathway in *Rhizobium leguminosarium* bv *trifolii*^38^. Malonic acid is activated before being broken down into acetate and carbon dioxide through decarboxylation^39^. It is often converted to malonyl-CoA by a CoA transferase, which is present in G22 (Phosphoribosyl-dephospho-CoA transferase) and the malonyl-CoA is then decarboxylated by malonate decarboxylase^35^,^39^. Malonate decarboxylase has four subunits; alpha, beta, gamma and delta; all of which are present in G22. The grassland isolate also contains two *mad* genes; *madL* and *madM.* These genes have previously been reported to be part of the malonate decarboxylase operon as transport proteins thought to be involved in malonate uptake^35^. The grassland isolate G22 also contains a malonyl CoA acyl carrier protein transacylase and triphosphoribosyl-dephospho-CoA synthetase. All genes involved in malonate decarboxylation are absent in the bare fallow isolate BF49. Malonate transport and utilisation genes are also present in other *Bradyrhizobium* isolates including BTAi1, ORS278, USDA 110, S23321 and all four of the forest strains (LTSP849, LTSP857, LTSP885 and LTSPM299). When grown in malonic acid, only G22 was able to metabolise it whereas BF49 and USDA 6 were not able to utilise this carbon source to the same extent.

The highest loadings and therefore the substrates which make the largest contribution to PC1 were L-pyroglutamic acid, L-leucine and D-galacturonic acid. L-pyroglutamic acid is an amino acid which is also known as 5-oxoproline. It is involved in the glutathione pathway and is converted to L-glutamic acid by the enzyme 5-oxoprolinase (EC 3.5.2.9)^40^,^41^. This gene is present in USDA 6 but is absent in both G22 and BF49. USDA 6 recorded a larger OD across all time points for this substrate than G22 and BF49. L-leucine is also an amino acid and is involved in numerous pathways including valine, leucine and isoleucine biosynthesis and degradation^40^,^41^. USDA 6 was able to utilise this substrate to a much greater extent than G22 and BF49 and the main difference in genes involved in L-leucine metabolism was the presence of leucine transaminase (EC 2.6.1.6) in USDA 6 and absence in G22 or BF49. D-galacturonic acid is a carbohydrate involved in numerous pathways including pentose and glucoronate interconversions, starch and sucrose metabolism and amino sugar and nucleotide sugar metabolism^40^,^41^. It is metabolised by USDA 6 more readily than G22 and BF49. This is likely due to the presence of pectinase (EC 3.2.1.15) and urinate dehydrogenase (EC 1.1.1.203) in USDA 6 but not in G22 or BF49. OD curves for the substrates discussed can be found in Supplementary Information S1.

### Plasmid

Plasmid replication genes *repABC* and *parAB* are only found on the G22 plasmid and not on either the G22 or BF49 chromosomes. The *trb* operon for conjugal transfer consists of 12 genes; *traI, trbBCDEJKLFGHI*^42^. From this operon, 10 out of the 12 genes are present on the G22 chromosome; only *trbH* and *traI* are missing. Conjugal transfer genes *trbBCDEFGIJL* are absent from BF49. Conjugative transfer DNA nicking endonuclease genes *traR*/*traO* are present only in the G22 plasmid. The other genes which were unique to the plasmid were two genes involved in purine utilisation (*yagS* and a putative xanthine transporter) and one for osmotic stress (*opgC*). The purine utilisation genes, *yagS*, encodes a periplasmic aromatic aldehyde oxidoreductase. YagS is usually part of an operon *yagTSRQ* which encodes a molybdenum-containing iron sulphur flavoprotein, where YagS is the FAD-containing subunit. The role of this protein has been suggested to be detoxification of aromatic aldehydes^43^,^44^. The flavoprotein produced from *yagTSRQ* shows homology with xanthine dehydrogenase^43^. The osmotic stress gene, *opgC*, is involved in the synthesis of osmoregulated periplasmic glucans (OPGs). The exact role of OPGs is not understood however they have been shown to potentially play a role in the interaction between bacteria and their eukaryotic host^45^,^46^. The OpgC protein has been examined in *Rhodobacter sphaeroides* and was shown to encode a succinyltransferase homolog involved in the succinyl modification of OPGs^45^,^46^. The other genes contained on the plasmid are also on the chromosome of both G22 and BF49 including genes for DNA ligases, DNA repair, cAMP signalling and RNA processing and modification.

## Conclusions

The strains described here are the first to be isolated and genome-sequenced from European soil and are unique compared to other completed *Bradyrhizobium* genomes due to the absence of previously characterised genes and gene clusters for symbiosis, nitrogen fixation and photosynthesis. They are also distinct from the North American forest isolates as G22 and BF49 contain genes for denitrification. They represent a major group, likely to play a key role in denitrification. The presence or absence of the terminal denitrification gene, *nosZ*, may determine whether the end product of denitrification is the potent greenhouse gas, nitrous oxide or the less problematic nitrogen gas. The carbon metabolism analysis shows that G22 and BF49 show different metabolic profiles over time and this are also distinct from a nodulating strain, USDA 6. The genomes and carbon metabolism analysis indicate that the free-living soil *Bradyrhizobium* have the potential to carry out many degradative and transformative functions in soil; the marked differences between two isolates from comparable soils that have undergone different management indicates that they form part of an extremely heterogeneous group.

## Methods

### Isolation and identification

*Bradyrhizobium* were isolated from the permanent grassland and bare fallow plots in the Highfield experiment at Rothamsted Research^12^. Serially diluted soil samples were plated onto modified arabinose gluconate (MAG) agar plates incubated at 28 °C^47^. Colonies forming after 7 days were picked, DNA extracted using MicroLYSIS-Plus using manufacturer’s instructions (Microzone, UK) and identified by PCR by the production of a 1360 bp 16S rRNA fragment using custom-designed *Bradyrhizobium* specific primers: Bradj16S70F (5′-GCGGGCGTAGCAATACGTCAGC-3′) and Bradj16S1430R (5′-GCCGGCTGCCTCCCTTGCGGGTTA-3′). Each PCR mixture (20 μl) consisted of 1x NH4 reaction buffer, 0.5 mM of each dNTP, 2.5 mM MgCl_2_, 0.1 μM of each primer, 1 U Biotaq polymerase (Bioline, UK) and 10 ng of genomic DNA. The PCR conditions were as follows: 95 °C 2 min, 30 cycles of 95 °C 15 sec, 68 °C 15 sec and 72 °C 1 min and extension at 72 °C for 10 min. The PCR products were examined on a 1.5% agarose gel stained with ethidium bromide (0.2 μg ml^−1^) at 100 V for 60 min. PCR products were purified using the Wizard SV gel and PCR clean up system (Promega, USA), sequenced as described in Mauchline *et al.* (2014)^48^ and identification confirmed using BLAST and searching the NCBI and RDP databases. Two isolates, one from grassland soil and one from bare fallow soil were chosen at random from the generated *Bradyrhizobium* culture collection to have their genomes sequenced *de novo.*

### De novo genome sequencing

DNA was extracted from isolates grown in MAG broth incubated at 28 °C and shaken at 100 rpm using the GenElute Bacterial Genomic DNA kit (Sigma Aldrich, USA). Extracted DNA was quantified using Qubit Fluorometric Quantification (Life Technologies, USA). Two sequencing platforms were used; Ion Torrent PGM™ (Life Technologies, USA) (located at Rothamsted Research, UK) and Illumina HiSeq 2000 (Illumina Inc, USA) (located at BGI, the Beijing Genomics Institute, China). For Ion Torrent sequencing, two barcoded, unamplified sequencing libraries were constructed using an Ion Plus Fragment Library Kit (Life Technologies, USA) using 1 μg template DNA. Libraries were pooled and sequence template generated using an Ion PGM™ Template OT2 400 Kit. Sequencing was performed using the Ion PGM™ Sequencing 400 Kit and an Ion 318™ Chip. The G22 sample generated 3,652,179 reads and the BF49 sample generated 3,129,252 reads. Illumina sequencing was performed by BGI and 40 ug of genomic DNA was used. A 6 kb mate library with 90 bp read length and 50X coverage was created using the Illumina HiSeq 2000 sequencing platform resulting in 2,997,699 paired reads for G22 and 3,026,005 paired reads for BF49.

### De novo genome assembly and annotation

The sequence data was assembled using SOAPdenovo2 assembler and gap closer^49^ using a maximum read length of 615 bp and the average insert size of 6 kb. A range of assemblies were produced using kmer values; 61–83, 81, 83, 85, 87, 89, 91. The genomes were manually curated using Geneious (Biomatters Ltd v8.1.5). Gap closing was carried out by PCR. Any remaining contigs from the 61–83 kmer reference assembly over 500 bp were investigated while those under were discarded. The G22 assembly had 19 contigs over 500 bp unplaced within the chromosome, with some containing annotated genes. No contigs over 500 bp remained after the completion of the final BF49 reference and so we can be confident that all genes are present in the assembly. The genomes were uploaded into RAST (Rapid Annotation using Subsystem Technology) for annotation^50^,^51^. The default parameters were used which were as follows: Classic RAST annotation scheme, RAST gene caller and Release70 FIGfam version. Automatically fix errors and backfill gaps were selected. The sequences have been deposited in the ENA database [Study ID: PRJEB10689, Sample ID G22 and unplaced contigs: ERS955657, G22 plasmid: ERS955536, Sample ID BF49: ERS954959]. The raw sequence data was also uploaded to ENA [G22: ERR1110561, BF49: ERR1110562] for Illumina and Ion Torrent [G22: ERR1110625, BF49: ERR1110597].

### Genome comparisons

Genome annotations were downloaded from RAST and examined manually using KEGG^40^,^41^. OrthoVenn was used to assess gene orthology using an e-value of 1e^−10^ 52. BRIG (BLAST Ring Image Generator) was used to compare the genomes with other *Bradyrhizobium* isolates (NCBI blast version 2.2.31)^53^. Large-Scale Genome Alignment Tool (LASTZ) was used to assess genome-wide sequence similarity in Geneious^54^.

### 16S rRNA phylogeny analysis

The 16S rRNA gene sequences for G22 and BF49 were compared with other Bradyrhizobium sequences in the NCBI database. All Bradyrhizobium 16S sequences from the NCBI RefSeq database and the four Bradyrhizobium isolates from North American forest soils^2^ were downloaded. These sequences were aligned using MUSCLE using 8 iterations^55^. The aligned region was extracted (1220 bp) and a phylogenetic tree was created using the neighbour joining clustering method with 1000 bootstraps. A 75% support threshold was used for drawing the phylogenetic tree. Accession numbers for the sequences used in this analysis can be found in Supplementary Information S2.

### Core genome phylogenetic analysis

OrthoVenn was used to identify genes which were present in all 9 genomes and were considered the “core genome”. The inflation value was set to 1.5 and an e-value of 1e^−10^. These genes were uploaded into the call SNPs and infer phylogeny (CSI) tool^56^ hosted by the Center for Genomic Epidemiology^57^ using the default options using *B. diazoefficiens* USDA 110 as the reference sequence. Default options were as follows: minimum of 10x depth at SNP positions, 10% relative minimum depth at SNP positions, a minimum of 10 bp distance between SNPs, minimum SNP quality score of 30, minimum read mapping quality score of 25 and minimum z-score of 1.96. Altered FastTree was selected. The core genome phylogenetic tree (Newick file) was visualised in Geneious.

### Biolog carbon assays

Isolates G22, BF49 and *B. japonicum* USDA 6 were grown in MAG broth, incubated at 28 °C and shaken at 100 rpm. Cell density was estimated from 1 ml of culture stained with 0.05% methylene blue using a haemocytometer. The cultures were diluted to a cell density of 10^6^ μl^−1^. The diluted cultures were centrifuged at 14000 × g for 1 minute, the supernatant was removed and the cells re-suspended in sterile deionised water. Each well of a Biolog GN2 MicroPlate™ was inoculated with 140 μl (10^8^ cells/ 140 μl) of bacterial culture. Three replicate plates per isolate were used. The optical density (OD) was read using a Varioscan SkanIt plate reader (Thermo Fisher Scientific Inc.) at 590 nm and 25 °C every 24 hours for a total of 98 hours (0, 24, 48, 72 and 98 hours). The plates were incubated at 25 °C and shaken at 100 rpm. Full list of substrates can be seen in Supplementary Information S3.

### Statistical analyses

The Biolog data was analysed using principal components analysis (PCA) using the inbuilt function, prcomp, in R (version 3.2.2). For visualisation of the PCA, biplots were drawn using PC1 and PC2 and the loadings matrix was extracted (see Supplementary Information S3). This identified specific substrates which were associated with the isolates and also substrates making the greatest contribution to the principal component. These pathways were then examined in the genomes. The substrates were grouped into carbohydrates, carboxylic acids, amines and amides, amino acids, polymers and miscellaneous according to categories identified in previously published work^58^.

## Additional Information

**How to cite this article**: Jones, F. P. *et al.* Novel European free-living, non-diazotrophic *Bradyrhizobium* isolates from contrasting soils that lack nodulation and nitrogen fixation genes – a genome comparison. *Sci. Rep.* **6**, 25858; doi: 10.1038/srep25858 (2016).

## Supplementary Material

Supplementary Information

Supplementary Information S3

## Figures and Tables

**Table 1:** Summary of the seven published complete *Bradyrhizobium* genomes and the two novel strains G22 and BF49.

Taxonomy	Strain	Host	Origin	Genome size (bp)	GC content	Proteins	rRNA operon	tRNA	Gene	Accession number	Reference
*Bradyrhizobium* sp.	BF49	Free-living	UK	7,547,693	63.80%	7380	1	48	7431	ERS954959	This paper
*Bradyrhizobium* sp.	G22	Free-living	UK	9,022,917	63.70%	8653	1	49	8706	ERS955657	This paper
*Bradyrhizobium* sp.	G22 plasmid	Free-living	UK	364,482	60.70%	541	1	2	546	ERS955536	This paper
*Bradyrhizobium* sp.	G22 unplaced	Free-living	UK	68,438	62.10%	81	0	0	81	ERS955657	This paper
*Bradyrhizobium* sp.	S23321	Free-living	Japan	7,231,841	64.30%	6898	1	45	6951	NC_017082	8
*B. diazoefficiens*	USDA 110	*Glycine max*	USA	9,105,828	64.10%	8317	1	50	8373	NC_004463	10
*B. japonicum*	USDA 6	*Glycine max*	Japan	9,207,384	63.70%	8829	2	51	8888	NC_017249	59
*B. japonicum*	E109	*Glycine max*	Argentina	9,224,208	63.70%	8233	2	54	8621	NZ_CP010313	60
*B. oligotrophicum*	S58	*Aeschynomene*	Japan	8,264,165	65.10%	7228	2	51	7285	NC_020453	11
*Bradyrhizobium* sp.	ORS278	*Aeschynomene*	Africa	7,456,587	65.50%	6752	2	50	6818	NC_009445	6
*Bradyrhizobium* sp.	BTAi1	*Aeschynomene*	N. America	8,264,687	64.90%	7394	2	52	7553	NC_009485	6
*Bradyrhizobium* sp.	BTAi1 plasmid	*Aeschynomene*	N. America	228,826	60.70%	203	0	0	216	NC_009475	6

**Table 2:** Summary of presence or absence of genes of interest in the seven published complete *Bradyrhizobium* genomes, four isolates from North America forest soils and the two novel strains G22 and BF49 (+ = present, − = absent).

Function	Gene(s)	G22	BF49	S23321	ORS278	BTAi1	S58	USDA 110	USDA 6	E109	>LTSP849	LTSP857	LTSP885	LTSPM299
Nitrogen fixation	*nifDKH*	−	−	+	+	+	+	+	+	+	−	−	−	−
Nitrogen fixation	*fixLJ*	+	+	+	+	+	+	+	+	+	−	−	−	−
Nodulation	*nodD*	−	−	−	−	−	−	+	+	+	−	−	−	−
Nodulation	*nodABC*	−	−	−	−	−	−	+	+	+	−	−	−	−
Denitrification	Nitrate reductase *napA/B*	+	+	+	+	+	+	+	+	+	+	+	+	+
Denitrification	Nitrite reductase *nirK*	+	+	+	+	+	+	+	+	+	−	−	−	−
Denitrification	Nitric oxide reductase *norB/C*	+	+	+	+	+	+	+	+	+	−	−	−	−
Denitrification	Nitrous oxide reductase *nosZ*	−	+	−	−	+	+	+	−	−	−	−	−	−
Uptake hydrogenase	*hup*	−	−	−	+	+	+	+	+	+	−	−	−	−
Photosynthesis	Bacteriochlorophyll *bchCXYZ/FNBHLM*	−	−	+	+	+	+	−	−	−	−	−	−	−
Photosynthesis	Carotenoids *crtEF*	−	−	+	+	+	+	−	−	−	−	−	−	−
Photosynthesis	LIght harvesting complexes *pucBAC/pufBA*	−	−	+	+	+	+	−	−	−	−	−	−	−
Photosynthesis	Reaction centre subunits *puhA/pufLM*	−	−	+	+	+	+	−	−	−	−	−	−	−
Carbon fixation	RuBisCo	+	+	+	+	+	+	+	+	+	+	+	+	+

## References

[b1] Uroz S., Buee M., Murat C., Frey-Klett P. & Martin F. Pyrosequencing reveals a contrasted bacterial diversity between oak rhizosphere and surrounding soil. Environ. Microbiol. Rep. 2, 281–288 (2010). 23766079 10.1111/j.1758-2229.2009.00117.x

[b2] Vaninsberghe D. *et al.* Non-symbiotic *Bradyrhizobium* ecotypes dominate North American forest soils. ISME J. 9, 2435–2441 (2015). 25909973 10.1038/ismej.2015.54 PMC4611507

[b3] Delmont T. O. *et al.* Structure, fluctuation and magnitude of a natural grassland soil metagenome. ISME J. 6, 1677–1687 (2012). 22297556 10.1038/ismej.2011.197 PMC3498926

[b4] Zhalnina K. *et al.* Ca. *Nitrososphaera* and *Bradyrhizobium* are inversely correlated and related to agricultural practices in long-term field experiments. Front. Microbiol. 4, 104–104 (2013). 23641242 10.3389/fmicb.2013.00104 PMC3640186

[b5] Van Rhijn P. & Vanderleyden J. The *Rhizobium*-plant symbiosis. Microbiol. Rev. 59, 124–142 (1995). 7708010 10.1128/mr.59.1.124-142.1995 PMC239357

[b6] Giraud E. *et al.* Legumes symbioses: Absence of *Nod* genes in photosynthetic bradyrhizobia. Science. 316, 1307–1312 (2007). 17540897 10.1126/science.1139548

[b7] Rivas R., Martens M., De Lajudie P. & Willems A. Multilocus sequence analysis of the genus *Bradyrhizobium*. Syst. Appl. Microbiol. 32, 101–110 (2009). 19201125 10.1016/j.syapm.2008.12.005

[b8] Okubo T. *et al.* Complete genome sequence of *Bradyrhizobium* sp S23321: Insights into symbiosis evolution in soil oligotrophs. Microbes Environ. 27, 306–315 (2012). 22452844 10.1264/jsme2.ME11321 PMC4036050

[b9] Sachs J. L., Ehinger M. O. & Simms E. L. Origins of cheating and loss of symbiosis in wild Bradyrhizobium. J. Evol. Biol. 23, 1075–1089 (2010). 20345811 10.1111/j.1420-9101.2010.01980.x

[b10] Kaneko T. *et al.* Complete genomic sequence of nitrogen-fixing symbiotic bacterium *Bradyrhizobium japonicum* USDA110. DNA Res. 9, 189–197 (2002). 12597275 10.1093/dnares/9.6.189

[b11] Okubo T. *et al.* Genome analysis suggests that the soil oligotrophic bacterium *Agromonas oligotrophica* (*Bradyrhizobium oligotrophicum*) is a nitrogen-fixing symbiont of *Aeschynomene indica*. Appl. Environ. Microbiol. 79, 2542–2551 (2013). 23396330 10.1128/AEM.00009-13 PMC3623176

[b12] Hirsch P. R. *et al.* Starving the soil of plant inputs for 50 years reduces abundance but not diversity of soil bacterial communities. Soil Biol. Biochem. 41, 2021–2024 (2009).

[b13] Weir B. S., Turner S. J., Silvester W. B., Park D. C. & Young J. A. Unexpectedly diverse *Mesorhizobium* strains and *Rhizobium leguminosarum* nodulate native legume genera of New Zealand, while introduced legume weeds are nodulated by *Bradyrhizobium* species. Appl. Environ. Microbiol. 70, 5980–5987 (2004). 15466541 10.1128/AEM.70.10.5980-5987.2004 PMC522066

[b14] Bejarano A., Ramirez-Bahena M.-H., Velazquez E. & Peix A. *Vigna unguiculata* is nodulated in Spain by endosymbionts of Genisteae legumes and by a new symbiovar (vignae) of the genus *Bradyrhizobium*. Syst. Appl. Microbiol. 37, 533–540 (2014). 24867807 10.1016/j.syapm.2014.04.003

[b15] Menna P., Barcellos F. G. & Hungria M. Phylogeny and taxonomy of a diverse collection of *Bradyrhizobium* strains based on multilocus sequence analysis of the 16S rRNA gene, ITS region and *glnII*, *recA*, *atpD* and *dnaK* genes. Int. J. Syst. Evol. Microbiol. 59, 2934–2950 (2009). 19628593 10.1099/ijs.0.009779-0

[b16] Menna P. & Hungria M. Phylogeny of nodulation and nitrogen-fixation genes in *Bradyrhizobium*: supporting evidence for the theory of monophyletic origin, and spread and maintenance by both horizontal and vertical transfer. Int. J. Syst. Evol. Microbiol. 61, 3052–3067 (2011). 21357454 10.1099/ijs.0.028803-0

[b17] Peix A. *et al.* Revision of the taxonomic status of the species *Rhizobium lupini* and reclassification as *Bradyrhizobium lupini* comb. nov. Int. J. Syst. Evol. Microbiol. 65, 1213–1219 (2015). 25609676 10.1099/ijs.0.000082

[b18] Ferrieres L., Francez-Charlot A., Gouzy J., Rouille S. & Kahn D. Fix-regulated genes evolved through promoter duplication in *Sinorhizobium meliloti*. Microbiology. 150, 2335–2345 (2004). 15256575 10.1099/mic.0.27081-0

[b19] Bobik C., Meilhoc E. & Batut J. FixJ: a major regulator of the oxygen limitation response and late symbiotic functions of *Sinorhizobium meliloti*. J. Bacteriol. 188, 4890–4902 (2006). 16788198 10.1128/JB.00251-06 PMC1482993

[b20] Meilhoc E., Cam Y., Skapski A. & Bruand C. The response to nitric oxide of the nitrogen-fixing symbiont *Sinorhizobium meliloti*. Mol. Plant. Microbe Interact. 23, 748–759 (2010). 20459314 10.1094/MPMI-23-6-0748

[b21] Delgado M. J., Bonnard N., Tresierra-Ayala A., Bedmar E. J. & Muller P. The *Bradyrhizobium japonicum napEDABC* genes encoding the periplasmic nitrate reductase are essential for nitrate respiration. Microbiology. 149, 3395–3403 (2003). 14663073 10.1099/mic.0.26620-0

[b22] Bedmar E. J., Robles E. F. & Delgado M. J. The complete denitrification pathway of the symbiotic, nitrogen-fixing bacterium *Bradyrhizobium japonicum*. Biochem. Soc. Trans. 33, 141–144 (2005). 15667287 10.1042/BST0330141

[b23] Fernandez L. A., Beatriz Perotti E., Antonio Sagardoy M. & Anahi Gomez M. Denitrification activity of *Bradyrhizobium* sp isolated from Argentine soybean cultivated soils. World J. Microbiol. Biotechnol. 24, 2577–2585 (2008).

[b24] Richardson D., Felgate H., Watmough N., Thomson A. & Baggs E. Mitigating release of the potent greenhouse gas N_2_O from the nitrogen cycle - could enzymic regulation hold the key? Trends Biotechnol. 27, 388–397 (2009). 19497629 10.1016/j.tibtech.2009.03.009

[b25] Henry S., Bru D., Stres B., Hallet S. & Philippot L. Quantitative detection of the *nosZ* gene, encoding nitrous oxide reductase, and comparison of the abundances of 16S rRNA, *narG*, *nirK*, and *nosZ* genes in soils. Appl. Environ. Microbiol. 72, 5181–5189 (2006). 16885263 10.1128/AEM.00231-06 PMC1538733

[b26] Shiina Y. *et al.* Relationship between soil type and N_2_O reductase genotype (*nosZ*) of indigenous soybean bradyrhizobia: *nosZ*-minus populations are dominant in andosols. Microbes Environ. 29, 420–426 (2014). 25476067 10.1264/jsme2.ME14130 PMC4262367

[b27] Zuber M., Harker A. R., Sultana M. A. & Evans H. J. Cloning and expression of *Bradyrhizobium japonicum* uptake hydrogenase structural genes in *Escherichia coli*. Proc. Natl. Acad. Sci. U. S. A. 83, 7668–7672 (1986). 3532119 10.1073/pnas.83.20.7668 PMC386782

[b28] Van Berkum P. Expression of uptake hydrogenase and hydrogen oxidation during heterotrophic growth of *Bradyrhizobium japonicum*. J. Bacteriol. 169, 4565–4569 (1987). 3115959 10.1128/jb.169.10.4565-4569.1987 PMC213822

[b29] Black L. K., Fu C. L. & Maier R. J. Sequences and characterization of *hupU* and *hupV* genes of *Bradyrhizobium japonicum* encoding a possible nickel-sensing complex involved in hydrogenase expression. J. Bacteriol. 176, 7102–7106 (1994). 7961478 10.1128/jb.176.22.7102-7106.1994 PMC197088

[b30] Gregor J. & Klug G. Regulation of bacterial photosynthesis genes by oxygen and light. FEMS Microbiol. Lett. 179, 1–9 (1999). 10481079 10.1111/j.1574-6968.1999.tb08700.x

[b31] Igarashi N. *et al.* Horizontal transfer of the photosynthesis gene cluster and operon rearrangement in purple bacteria. J. Mol. Evol. 52, 333–341 (2001). 11343129 10.1007/s002390010163

[b32] Gourion B. *et al.* Bacterial RuBisCO is required for efficient *Bradyrhizobium*/*Aeschynomene* symbiosis. PLoS One. 6, e21900 (2011). 21750740 10.1371/journal.pone.0021900 PMC3130060

[b33] Horecker B. L., Smyrniotis P. Z. & Hurwitz J. Role of xylulose 5-phosphate in the transketolase reaction. J. Biol. Chem. 223, 1009–1019 (1956). 13385248

[b34] Fullam E., Pojer F., Bergfors T., Jones T. A. & Cole S. T. Structure and function of the transketolase from Mycobacterium tuberculosis and comparison with the human enzyme. Open Biol. 2, 110026 (2012). 22645655 10.1098/rsob.110026 PMC3352088

[b35] Dimroth P. & Hilbi H. Enzymic and genetic basis for bacterial growth on malonate. Mol. Microbiol. 25, 3–10 (1997). 11902724 10.1046/j.1365-2958.1997.4611824.x

[b36] Kim Y. S. Malonate metabolism: Biochemistry, molecular biology, physiology, and industrial application. J. Biochem. Mol. Biol. 35, 443–451 (2002). 12359084 10.5483/bmbrep.2002.35.5.443

[b37] Suvorova I. A., Ravcheev D. A. & Gelfand M. S. Regulation and Evolution of Malonate and Propionate Catabolism in Proteobacteria. J. Bacteriol. 194, 3234–3240 (2012). 22505679 10.1128/JB.00163-12 PMC3370838

[b38] Kim Y. S., Kwon S. J. & Kang S. W. Malonyl-CoA synthetase from *Rhizobium trifolii:* Purification, properties, and the immunological comparison with those from *Bradyrhizobium japonicum* and *Pseudomonas fluorescens*. *Korean* Biochem. J. 26, 176–183 (1993).

[b39] Schmid M., Berg M., Hilbi H. & Dimroth P. Malonate decarboxylase of *Klebsiella pneumoniae* catalyses the turnover of acetyl and malonyl thioester residues on a coenzyme-A-like prosthetic group. Eur. J. Biochem. 237, 221–228 (1996). 8620876 10.1111/j.1432-1033.1996.0221n.x

[b40] Kanehisa M. & Goto S. KEGG: Kyoto Encyclopedia of Genes and Genomes. Nucleic Acids Res. 28, 27–30 (2000). 10592173 10.1093/nar/28.1.27 PMC102409

[b41] Kanehisa M., Sato Y., Kawashima M., Furumichi M. & Tanabe M. KEGG as a reference resource for gene and protein annotation. Nucleic Acids Res. 44, D457–D462 (2016). 26476454 10.1093/nar/gkv1070 PMC4702792

[b42] Li P. L., Hwang I., Miyagi H., True H. & Farrand S. K. Essential components of the Ti plasmid trb system, a type IV macromolecular transporter. J. Bacteriol. 181, 5033–5041 (1999). 10438776 10.1128/jb.181.16.5033-5041.1999 PMC93993

[b43] Neumann M. *et al.* A periplasmic aldehyde oxidoreductase represents the first molybdopterin cytosine dinucleotide cofactor containing molybdo-flavoenzyme from *Escherichia coli*. Febs Journal. 276, 2762–2774 (2009). 19368556 10.1111/j.1742-4658.2009.07000.x

[b44] Xi H. L., Schneider B. L. & Reitzer L. Purine catabolism in *Escherichia coli* and function of xanthine dehydrogenase in purine salvage. J. Bacteriol. 182, 5332–5341 (2000). 10986234 10.1128/jb.182.19.5332-5341.2000 PMC110974

[b45] Kawaharada Y., Kiyota H., Eda S., Minamisawa K. & Mitsui H. Identification of the *Mesorhizobium loti* gene responsible for glycerophosphorylation of periplasmic cyclic beta-1,2-glucans. FEMS Microbiol. Lett. 302, 131–137 (2010). 19951365 10.1111/j.1574-6968.2009.01843.x

[b46] Roset M. S., Ciochini A. E., Ugalde R. A. & De Iannino N. I. The *Brucella abortus* cyclic beta-1,2-glucan virulence factor is substituted with O-ester-linked succinyl residues. J. Bacteriol. 188, 5003–5013 (2006). 16816173 10.1128/JB.00086-06 PMC1539967

[b47] Van Berkum P. Evidence for a third uptake hydrogenase phenotype among the soybean bradyrhizobia. Appl. Environ. Microbiol. 56, 3835–3841 (1990). 16348383 10.1128/aem.56.12.3835-3841.1990 PMC185076

[b48] Mauchline T. H., Hayat R., Roberts R., Powers S. J. & Hirsch P. R. Assessment of core and accessory genetic variation in *Rhizobium leguminosarum* symbiovar *trifolii* strains from diverse locations and host plants using PCR-based methods. Lett. Appl. Microbiol. 59, 238–246 (2014). 24739023 10.1111/lam.12270

[b49] Luo R. *et al.* SOAPdenovo2: an empirically improved memory-efficient short-read *de novo* assembler. GigaScience. 1, 18–18 (2012). 23587118 10.1186/2047-217X-1-18 PMC3626529

[b50] Aziz R. K. *et al.* The RAST Server: rapid annotations using subsystems technology. BMC Genomics. 9, 75 (2008). 18261238 10.1186/1471-2164-9-75 PMC2265698

[b51] Overbeek R. *et al.* The SEED and the Rapid Annotation of microbial genomes using Subsystems Technology (RAST). Nucleic Acids Res. 42, D206–D214 (2014). 24293654 10.1093/nar/gkt1226 PMC3965101

[b52] Wang Y., Coleman-Derr D., Chen G. & Gu Y. Q. OrthoVenn: a web server for genome wide comparison and annotation of orthologous clusters across multiple species. Nucleic Acids Res. 43, W78–W84 (2015). 25964301 10.1093/nar/gkv487 PMC4489293

[b53] Alikhan N. F., Petty N. K., Ben Zakour N. L. & Beatson S. A. BLAST Ring Image Generator (BRIG): simple prokaryote genome comparisons. BMC Genomics. 12, 402 (2011). 21824423 10.1186/1471-2164-12-402 PMC3163573

[b54] Harris R. S. *Improved pairwise alignment of genomic DNA.* PhD, The Pennsylvania State University. (2007).

[b55] Edgar R. C. MUSCLE: a multiple sequence alignment method with reduced time and space complexity. BMC Bioinformatics. 5, 1–19 113 (2004). 15318951 10.1186/1471-2105-5-113 PMC517706

[b56] Kaas R. S., Leekitcharoenphon P., Aarestrup F. M. & Lund O. Solving the problem of comparing whole bacterial genomes across different sequencing platforms. PLoS One. 9, e104984 (2014). 25110940 10.1371/journal.pone.0104984 PMC4128722

[b57] *Center for Genomic Epidemiology*. (2011) Available at: http://www.genomicepidemiology.org/. (Accessed: 11th November 2015).

[b58] Preston-Mafham J., Boddy L. & Randerson P. F. Analysis of microbial community functional diversity using sole-carbon-source utilisation profiles - a critique. FEMS Microbiol. Ecol. 42, 1–14 (2002). 19709261 10.1111/j.1574-6941.2002.tb00990.x

[b59] Kaneko T. *et al.* Complete genome sequence of the soybean symbiont *Bradyrhizobium japonicum* strain USDA6^T^. Genes. 2, 763–787 (2011). 24710291 10.3390/genes2040763 PMC3927601

[b60] Torres D. *et al.* Genome sequence of *Bradyrhizobium japonicum* E109, one of the most agronomically used nitrogen-fixing rhizobacteria in Argentina. Genome announcements. 3 (2015). 10.1128/genomeA.01566-14 PMC4335331 25700406

