# Supplementary material for: Novel European free-living, non-diazotrophic Bradyrhizobium isolates from contrasting soils that lack nodulation and nitrogen fixation genes – a genome comparison
Source: Sci Rep. 2016 May 10;6:25858. doi: 10.1038/srep25858 (PMC4861915; doi:10.1038/srep25858)
Supplement: Supplementary Information [file srep25858-s1.pdf]

Supplementary Information

Novel European free-living, non-diazotrophic *Bradyrhizobium* isolates from contrasting soils that lack nodulation and nitrogen fixation genes – a genome comparison

Frances Patricia Jones*[1, 3], Ian M. Clark[1], Robert King[2], Liz J. Shaw[3], Martin J. Woodward[4] and Penny R. Hirsch[1]

[1]Department of AgroEcology, Rothamsted Research, Harpenden, AL5 2JQ, UK

[2]Department of Computational and Systems Biology, Rothamsted Research, Harpenden, AL5 2JQ, UK

[3]Department of Geography and Environmental Science, University of Reading, Reading, RG6 6AH, UK

[4]Department of Food and Nutritional Sciences, University of Reading, Reading, RG6 6AH, UK

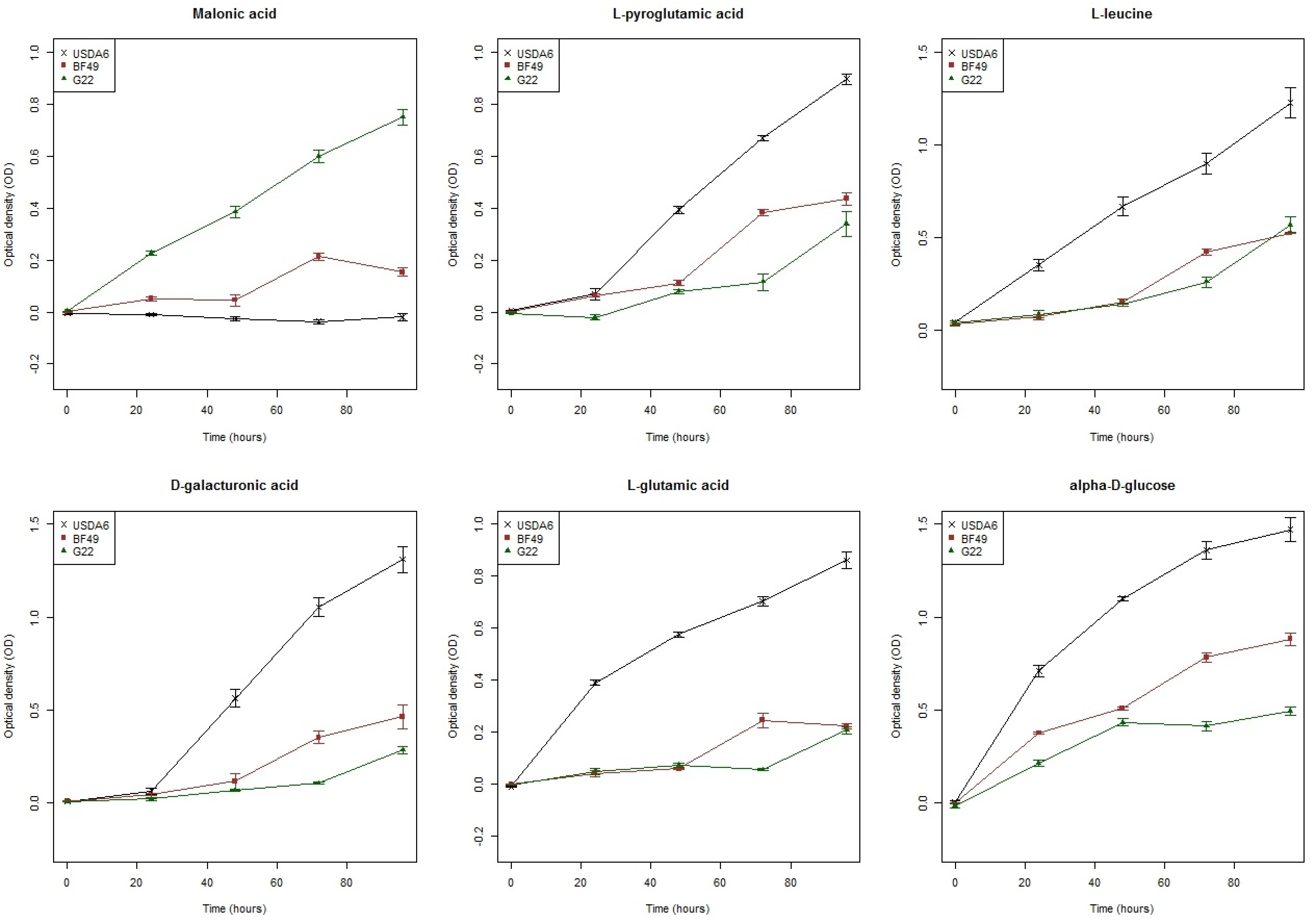

Supplementary Information S1: Carbon metabolism curves for substrates with close association with G22 and the highest overall loadings for PC1.

Supplementary Information S2: Accession number for sequences used in 16S phylogeny

	Sequence name	Sequence length	Accession number
1	Bradyrhizobium neotropicale strain BR 10247	1301 bp	NR_133987.1
2	Bradyrhizobium icense strain LMTR 13	1413 bp	NR_133707.1
3	Bradyrhizobium oligotrophicum strain S58	1484 bp	NR_118384.1
4	Bradyrhizobium rifense strain CTAW71	1516 bp	NR_116361.1
5	Bradyrhizobium pachyrhizi strain PAC48	1483 bp	NR_043037.1
6	Bradyrhizobium jicamae strain PAC68	1484 bp	NR_043036.1
7	Bradyrhizobium ottawaense strain OO99	1420 bp	NR_133988.1
8	Bradyrhizobium manausense strain BR 3351	1353 bp	NR_133986.1
9	Bradyrhizobium ingae strain BR 10250	1358 bp	NR_133985.1
10	Bradyrhizobium ganzhouense strain RITF806	1363 bp	NR_133706.1
11	Bradyrhizobium valentinum strain LmjM3	1490 bp	NR_125638.1
12	Bradyrhizobium elkanii strain USDA 76	1340 bp	NR_117947.1
13	Bradyrhizobium daqingense strain CCBAU 15774	1300 bp	NR_117944.1
14	Bradyrhizobium arachidis strain CCBAU 051107	1347 bp	NR_117791.1
15	Bradyrhizobium lablabi strain CCBAU 23086	1356 bp	NR_117513.1
16	Bradyrhizobium cytisi strain CTAW11	1516 bp	NR_116360.1
17	Bradyrhizobium yuanmingense strain B071	1426 bp	NR_028768.1
18	Bradyrhizobium paxllaeri strain LMTR 21	1413 bp	NR_133708.1
19	Bradyrhizobium huanghuaihaiense strain CCBAU 23303	1348 bp	NR_117945.1
20	Bradyrhizobium liaoningense strain 2281	1336 bp	NR_114611.1
21	Bradyrhizobium canariense strain BTA-1	1481 bp	NR_042177.1
22	Bradyrhizobium lupini strain USDA 3051	1480 bp	NR_134836.1
23	Bradyrhizobium denitrificans strain LMG 8443	1443 bp	NR_118982.1
24	Bradyrhizobium japonicum strain LMG 6138	1441 bp	NR_118981.1
25	Bradyrhizobium denitrificans strain LMG 8443	1443 bp	NR_118770.1
26	Bradyrhizobium daqingense strain CCBAU 15774	1372 bp	NR_118648.1
27	Bradyrhizobium retamae strain Ro19	1345 bp	NR_118548.1
28	Bradyrhizobium liaoningense strain LMG 18230	1430 bp	NR_114733.1
29	Bradyrhizobium japonicum strain NBRC 14783	1414 bp	NR_112930.1
30	Bradyrhizobium liaoningense strain NBRC 100396	1414 bp	NR_112929.1
31	Bradyrhizobium yuanmingense strain NBRC 100594	1414 bp	NR_112928.1
32	Bradyrhizobium elkanii strain NBRC 14791	1414 bp	NR_112927.1
33	Bradyrhizobium iriomotense strain EK05	1413 bp	NR_112671.1
34	Bradyrhizobium liaoningense strain 2281	1425 bp	NR_112095.1
35	Bradyrhizobium liaoningense strain 2281	1476 bp	NR_041785.1
36	Bradyrhizobium elkanii strain USDA 76	1480 bp	NR_036953.1
37	Bradyrhizobium japonicum strain 3I1b6	1481 bp	NR_036865.1
38	Bradyrhizobium betae strain PL7HG1	1483 bp	NR_029104.1
39	Bradyrhizobium japonicum strain DSM 30131	1482 bp	NR_119191.1
40	Bradyrhizobium elkanii strain ATCC 49852	1042 bp	NR_114610.1
41	Bradyrhizobium canariense strain NBRC 103049	1414 bp	NR_114199.1
42	Bradyrhizobium betae strain NBRC 103048	1414 bp	NR_114198.1
43	Bradyrhizobium iriomotense strain NBRC 102520	1414 bp	NR_114138.1
44	Bradyrhizobium denitrificans strain IFAM 1005	1477 bp	NR_041827.1
45	Bradyrhizobium sp. LTSP849	1497 bp	NZ_JYMR01000018.1
46	Bradyrhizobium sp. LTSP857	1497 bp	NZ_JYMS01000032.1
47	Bradyrhizobium sp. LTSP885	1499 bp	NZ_JYMT01000017.1
48	Bradyrhizobium sp. LTSPM299	1499 bp	NZ_JYMU01000004.1

49	Bradyrhizobium sp. S23321	1490 bp	NC_017082
50	B. diazoefficiens USDA 110	1490 bp	NC_004463
51	B. japonicum USDA 6	1490 bp	NC_017249
52	B. japonicum E109	1498 bp	NZ_CP010313
53	B. oligotrophicum S58	1493 bp	NC_020453
54	Bradyrhizobium sp. ORS278	1489 bp	NC_009445
55	Bradyrhizobium sp. BTAi1	1489 bp	NC_009485
